# IMPLEMENTATION OF WALANT ANESTHESIA FOR HAND AND WRIST SURGERIES IN A TERTIARY HOSPITAL: PILOT STUDY

**DOI:** 10.1590/1413-785220253303e289446

**Published:** 2025-08-18

**Authors:** Lucas Moratelli, Maria Teresa Cursino Moratelli, Marcos Felipe Marcatto de Abreu, João Carlos Nakamoto, Rodrigo Gonçalves Pagnano

**Affiliations:** 1 Universidade Estadual de Campinas (UNICAMP), Faculdade de Ciencias Medicas, Departamento de Ortopedia, Reumatologia e Traumatologia, Campinas, SP, Brazil.; 2 Universidade Estadual de Campinas (UNICAMP), Faculdade de Ciencias Medicas, Departamento de Anestesiologia, Oncologia e Radiologia, Campinas, SP, Brazil.; 3 Instituto Vita, São Paulo, SP, Brazil.

**Keywords:** Anesthesia, Local, Epinephrine, Ambulatory Surgical Procedures, Anestesia Local, Epinefrina, Procedimentos Cirúrgicos Ambulatórios

## Abstract

**Objective:**

To analyze the feasibility and patient satisfaction of using the WALANT technique in treating low-complexity hand and wrist surgical conditions.

**Methods:**

A prospective pilot study conducted in a tertiary hospital, including individuals aged ≥18 years, ASA I or II, undergoing low-complexity procedures lasting <1 hour. Those with contraindications to local anesthesia or clinical conditions that could affect the study data were excluded. Participants received WALANT anesthesia. Demographic and clinical data were collected, and the Numerical Pain Scale measured pain.

**Results:**

Twenty-one participants who underwent 23 surgical procedures were analyzed. 69.6% were women, a mean age of 49.6 ± 15.6 years, and 73.9% were ASA II. During the procedures, 86.5% reported tolerable pain only once, attributed to the needle puncture of the skin (mean of 2.0 ± 1.7 on a scale of 0-10). Only 5% reported that the pain of anesthesia was greater than that of contralateral venous access. Intraoperative bleeding was tolerable.

**Conclusion:**

The WALANT technique showed sufficient anesthetic and vasoconstrictor efficacy for most low-complexity hand and wrist surgeries and ensured patient comfort even without sedation. ***Level of Evidence IV; Case Series***.

## RESUMO

**Objetivo:**

Analisar a viabilidade e a satisfação dos pacientes usando a técnica WALANT no tratamento das afecções cirúrgicas de baixa complexidade da mão e do punho.

**Métodos:**

Estudo piloto prospectivo realizado em hospital terciário, incluindo indivíduos com idade ≥18 anos, ASA I ou II, submetidos a procedimentos de baixa complexidade com duração <1 hora. Foram excluídos aqueles com contraindicações à anestesia local ou condições clínicas que pudessem afetar os dados do estudo. Todos os participantes receberam anestesia WALANT. Dados demográficos e clínicos foram coletados, e a dor foi medida pela Escala Numérica de dor.

**Resultados:**

Analisados 21 participantes submetidos a 23 procedimentos cirúrgicos. 69,6% eram mulheres, média de idade 49,6 ± 15,6 anos, e 73,9% ASA II. Durante os procedimentos, 86,5% relataram dor tolerável apenas uma vez, atribuída à perfuração da pele pela agulha (média de 2,0 ± 1,7 em escala de 0-10). Apenas 5% relataram que a dor da anestesia foi superior à dor do acesso venoso contralateral. O sangramento intraoperatório foi tolerável.

**Conclusão:**

A técnica WALANT apresentou eficácia anestésica e vasoconstritora suficiente para a maior parte das cirurgias de baixa complexidade da mão e do punho e garantiu o conforto dos pacientes mesmo sem o uso de sedação. ***Nível de Evidência IV; Série de Casos.***

## INTRODUCTION

WALANT (Wide-awake Local Anesthesia No Tourniquet) is a local anesthesia technique that uses a local anesthetic combined with a vasoconstrictor, eliminating the need for a tourniquet. Lalonde et al. clarified the safety of epinephrine use in local anesthetics in a study of 3,110 cases of hand surgery using 1% lidocaine with epinephrine 1:100,000, without identifying cases of clinically relevant digital ischemia or necrosis, or the need for the antidote, phentolamine.^1^

In Brazil, Novais Junior et al. and Barros et al. were pioneers in publishing studies on the WALANT technique.^2-4^ Sardenberg et al. presented a study with 488 cases, confirming the safety of the technique and highlighting regional particularities, such as the production of the anesthetic solution in concentrations not commercially available in the country.^5^ Recently, Alves et al. emphasized the benefits of the technique in the face of the Covid-19 pandemic.^6^

This study aimed to analyze the feasibility of implementation and patient satisfaction using the WALANT technique in the treatment of low-complexity surgical conditions of the hand and wrist in a tertiary hospital.

## METHODS

This is a pilot study conducted as a prospective case series at a tertiary hospital. Approval was obtained from the local research ethics committee (CAAE 95576418.7.0000.5404).

Individuals aged 18 years or older classified as ASA (American Society of Anesthesiologists) I or II undergoing low-complexity hand, wrist, or elbow procedures with an estimated duration of less than 1 hour were included. Those who presented contraindications to the use of local anesthesia, such as refusal (desire to fall asleep) and presence of infections at the injection site, were excluded. Those with previous diseases in the same limb that could influence the outcome of the study (e.g., any previous sensory changes, cervicobrachialgia, complex regional pain syndrome, or other chronic pain unrelated to surgical treatment), conditions with an increased risk of tissue hypoperfusion (e.g., scleroderma), and surgeries with a potential risk of compartment syndrome. Fractures older than 21 days were also excluded from the study.

Participants underwent WALANT anesthesia according to the research protocol that follows the original technique described by Lalonde, but adapted to the characteristics of the service. The injection sites for the anesthetic and the volumes used depended on the surgery the patient underwent. (Table 1)

**Table 1:** Volumes of anesthetic and injection sites used.

Procedure	Volume total	Injection volumes and sites
Carpal tunnel syndrome	20 ml	10 ml between the median and ulnar nerves 10 ml below the incision
Trigger finger	4 ml	4 ml subcutaneously below the incision
Total digital lock (arthrodesis IF, nail bed, fingertips)	8 ml	2 ml applied to four points: midvolar and middorsal on the proximal and middle phalanges
Dupuytren	15 ml/ radius	10 ml in the palm of your hand 2 ml under each proximal and middle phalanx 1 ml under the distal phalanx if necessary
Stener's lesion	15 ml	2 ml in the proximal phalanx 2 ml dorsally in the proximal phalanx Remaining around the metacarpal phalangeal joint
Cubital tunnel syndrome	40 ml	20 ml proximal to the start of the incision 20 ml under the incision (spinal needle)

IF: interphalangeal joints.

A solution of 1% lidocaine with epinephrine at 1:100,000 was used, plus 8.4% sodium bicarbonate in a 1:10 ratio. Whenever possible, participants were anesthetized in a preparation room at least 30 minutes before the incision to ensure maximum effect of the vasoconstrictor. Vital signs were obtained during patient preparation, at the time of anesthesia administration, and every 10 minutes after anesthesia. Intraoperative pain or discomfort at the incision site (verbal pain scale from 0 to 10, corresponding to increasing pain intensity), pain or discomfort related to the administration of anesthesia or intraoperative positioning (verbal pain scale, reports of intolerance to the local anesthetic, reports of discomfort in the lower limbs or contralateral upper limb associated with positioning), degree of intraoperative bleeding and difficulty in visualizing the surgical field (need for additional cauterization after initial standard approach, need for tourniquet), need for sedatives (Midazolam); presence of complications related to the anesthetic (neurotoxicity: metallic taste, agitation, lethargy, paresthesias, convulsions), presence of complications associated with the sedative (respiratory depression, *delirium*), and presence of complications related to the vasoconstrictor (hypoperfusion of the extremities after the procedure). During the postoperative follow-up, participants were asked about their satisfaction with the method and, if they needed a similar procedure, whether they would choose the same anesthesia or prefer to be put to sleep. In the first three weeks after surgery, signs of infection were observed.

The data were evaluated according to frequency or measures of central tendency and dispersion. The data were calculated using IBM® SPSS® Statistics version 24.0 for Windows software (Armonk, NY, USA), assuming a significance level of 5%.

## RESULTS

The protocol was applied to 21 participants who underwent 23 surgical procedures on the hand, wrist, and elbow. (Table 2)

**Table 2:** Procedures performed.

Procedures	n	%
Carpal tunnel syndrome	6	26.1%
Trigger finger (II to V)	6	26.1%
Trigger finger	2	8.7%
Dupuytren	2	8.7%
Amputation at the metacarpophalangeal joint	1	4.3%
Glomus tumor	1	4.3%
Stener's lesion	1	4.3%
Arthrodesis of the IFP joint	1	4.3%
Dorsal synovial cyst resection	1	4.3%
Resection of flexor pollicis cyst	1	4.3%
Cubital tunnel syndrome (elbow)	1	4.3%

Female participants accounted for 69.6%. The mean age was 49.6 ± 15.6 years, and the physical status classification according to the ASA (American Society of Anesthesiologists) scale I and II was 23.1% and 73.9%, respectively. All procedures were elective, with 91.3% involving only soft tissue manipulation and 8.7% involving bone manipulation. (Table 3)

**Table 3:** Patient and procedure data.

Variable	n	%
Age (years)	49.6 ±	15.6*
**Sex**		
Male	7/23	(30.4%)
Women	16/23	(69.6%)
**ASA classification**		
ASA I	5/23	(26.1%)
ASA II	17/23	(73.9%)
**Procedure**		
Soft parts	21/23	(91.3%)
Bones	2/23	(8.7%)
**Nature of the surgery**		
Elective	23/23	(100%)
**Prophylactic antibiotic**		
Start of anesthesia	23/23	(100%)

* mean ± standard deviation.

As for pain during the anesthetic and surgical procedures, 87.0% of participants reported feeling pain only once during the entire period, which was attributed to the first penetration of the needle into the skin (mean of 2.0 ± 1.7 points on a scale of 0-10). No participant reported pain during instillation of the anesthetic.

The remaining 13.0% reported experiencing a second episode of pain (mean 3.0 ± 1.4 points on a scale of 0-10), with 4.3% attributing it to the administration of anesthesia and 8.7% to intraoperative pain. The procedures that generated reports of intraoperative pain involved manipulation of deep structures that did not correspond to the site of the lesions, with one case involving trigger finger surgery and another involving a Stener lesion.

In comparison, the average pain score reported during peripheral venous access in the antecubital region of the contralateral upper limb was 3.8 ± 2.3 points. Only 5% of participants considered the pain of anesthesia administration to be greater than the pain felt during peripheral venous access.

Epinephrine was effective in controlling bleeding in most procedures, except for glomus tumor removal and selective Dupuytren fasciotomies. The glomus tumor surgery presented slight bleeding in absolute values, but it was enough to make the procedure unfeasible and required the use of a digital tourniquet. In the two fasciectomies performed to treat Dupuytren's contractures, there was continuous bleeding in small amounts, but it was possible to complete the surgeries with good visualization of the neurovascular bundles, and a tourniquet was not necessary.

Twelve patients were anesthetized in the preparation room before entering the operating room. The mean preoperative (time between admission to the operating room and surgical incision) and postoperative (time between dressing and discharge from the operating room) non-surgical times were 11.8 ± 2.5 minutes and 5.1 ± 1.3 minutes, respectively.

No clinical complications were identified. A patient presented with symptoms of anxiety and sinus tachycardia, which were promptly controlled with intravenous administration of 2 mg of Midazolam. Three weeks after surgery, all participants showed good healing. No ischemic complications, infections, or complex regional pain syndrome were identified.

All patients reported satisfaction with WALANT anesthesia. When asked if they would undergo local anesthesia again if they needed a similar surgery, 95.2% would repeat the procedure with WALANT and 4.8% would prefer to be put to sleep.

## DISCUSSION

The WALANT method eliminates the need for sedation because it ensures adequate anesthesia and hemostasis at the surgical site without the use of a tourniquet. Throughout the procedure, the only painful stimulus felt by 86.5% of participants was the initial needle puncture of the skin, and this pain was reported as tolerable. A study analyzing the training of resident physicians in the use of the WALANT method showed that 76% of patients felt pain only during the first injection, and that in 88% of cases, the VAS score was less than or equal to 1.^7^ This reveals that even surgeons with no experience with the WALANT method can achieve similar results.

At the time of the study, the 1% lidocaine solution with epinephrine 1:100,000 was not commercially available in Brazil, and some dilutions were proposed. In the Brazilian study conducted by Sardenberg et al., the solution was prepared in a simple and more understandable way by mixing 0.2 ml of epinephrine (1 mg/ml) with 20 ml of 1% lidocaine; sodium bicarbonate was not included in order to reduce the number of dilutions and the likelihood of errors in production.^5^ In the present study, we chose to compose the final solution with 8.4% sodium bicarbonate in a 1:10 ratio to neutralize the pH and reduce the burning sensation characteristic of the acid injection of lidocaine. In addition to adjusting the pH using sodium bicarbonate, other strategies to minimize pain at the injection site were included in the protocol, including the use of a fine needle.^8-11^ In his book, Lalonde recommends the use of 27G to 30G needles.^12^ However, the thinnest needle available in the service was 13 × 0.45 mm (26G ½), which is slightly thicker and shorter.

Many advantages have been reported with the use of the WALANT technique, one of which is the possibility of dispensing with preoperative examinations. In general, the literature is clear in considering that the indication for preoperative tests should be individualized based on clinical criteria and according to the procedure to be performed.^13^ Davison et al. observed that, in patients undergoing carpal tunnel syndrome surgery, additional tests were requested for 48% of patients operated on with sedation and for 3% of patients operated on with WALANT, demonstrating that most preoperative tests were requested because of sedation and not because of surgery.^14^

Some procedures presented bleeding above the tolerable level. In the case of glomus tumor removal, minimal and constant bleeding in the nail bed prevented correct identification of the nail bed and tumor, which is why a digital tourniquet was applied. We believe that, because the glomus tumor was subungual and we administered anesthesia before removing the nail, the epinephrine was not effectively deposited below the incision. Although the tourniquet was necessary, it did not cause any discomfort because the finger was completely blocked. In view of this, we conclude that the WALANT technique would not be the most appropriate for removing the glomus tumor, recommending digital blockage with a tourniquet at the base of the finger, ensuring a clean surgical field and adequate visualization of the nail bed by the surgeon, without compromising patient comfort.

No cases of hypoperfusion or digital necrosis occurred in this series. Although the safety of the technique has been demonstrated by many studies, some complications have been reported with the popularization of the method and its widespread use.

scale. However, clinically severe cases have been observed in places where phentolamine was not available, which is why its availability is recommended.^15,16^ In addition, a case of venous congestion was reported in the literature during the treatment of a distal radius fracture with WALANT, but it resulted from the injection of a large volume of anesthetic and not from vasoconstriction. Full recovery in this case was observed after a few weeks.^17^

In this series, preoperative antibiotic prophylaxis was administered to all participants, and there were no cases of postoperative infection after 3 weeks. In elective hand and wrist surgeries, the need for antibiotic prophylaxis is a matter of debate.^18^ There are recommendations that prophylaxis may be unnecessary in clean elective surgeries lasting up to 2 hours in patients without comorbidities and without the use of implants.^19^ On the other hand, Sandrowski et al. studied the use of a single dose of Cefazolin or Clindamycin as preoperative prophylaxis in outpatient hand surgeries and identified low rates of adverse effects related to antibiotics, the main ones being skin rash (0.9%) and diarrhea (0.5%).^20^

This study has some limitations. This is a pilot study with a small number of cases, which is why we are seeking to increase the variety of procedures tested. In addition, patients classified as ASA III and IV were excluded, for whom the literature points to the WALANT technique as a good alternative to other anesthetic modalities due to the lower incidence of complications. Furthermore, the data were collected by the same researcher who administered the anesthesia, which may contain biases associated with the lack of blinding, and there was no comparison with other anesthetic methods. However, it was possible to establish the feasibility of applying the technique in a tertiary public hospital, which may contribute to an increase in the number of less complex hand and wrist procedures.

## CONCLUSION

The WALANT technique demonstrated sufficient anesthetic and vasoconstrictive efficacy for most low-complexity hand and wrist surgeries and ensured patient comfort even without the use of sedation.
